# Novel Therapies for Cardiometabolic Disease: Recent Findings in Studies with Hormone Peptide-Derived G Protein Coupled Receptor Agonists

**DOI:** 10.3390/nu14183775

**Published:** 2022-09-13

**Authors:** Elena Jiménez-Martí, Gema Hurtado-Genovés, María Aguilar-Ballester, Sergio Martínez-Hervás, Herminia González-Navarro

**Affiliations:** 1 Department of Biochemistry and Molecular Biology, Faculty of Medicine, University of Valencia, 46010 Valencia, Spain; 2 Institute of Health Research INCLIVA, 46010 Valencia, Spain; 3 Centro de Investigación Biomédica en Red de Cáncer, 28029 Madrid, Spain; 4 Department of Medicine, Faculty of Medicine, University of Valencia, Endocrinology and Nutrition Service, Universitary Clinic Hospital of Valencia, 46010 Valencia, Spain; 5 Centro de Investigación Biomédica en Red de Diabetes y Enfermedades Metabólicas Asociadas, 28029 Madrid, Spain

**Keywords:** type 2 diabetes mellitus, incretins, glucagon, cardiometabolism, GPCR agonism, clinical trial

## Abstract

The increasing prevalence of obesity and type 2 diabetes (T2DM) is provoking an important socioeconomic burden mainly in the form of cardiovascular disease (CVD). One successful strategy is the so-called metabolic surgery whose beneficial effects are beyond dietary restrictions and weight loss. One key underlying mechanism behind this surgery is the cooperative improved action of the preproglucagon-derived hormones, glucagon, glucagon-like peptide-1 (GLP-1), and glucose-dependent insulinotropic polypeptide (GIP) which exert their functions through G protein-coupled receptors (GPCR). Great success has been reached with therapies based on the GLP-1 receptor monoagonism; therefore, a logical and rational approach is the use of the dual and triagonism of GCPC to achieve complete metabolic homeostasis. The present review describes novel findings regarding the complex biology of the preproglucagon-derived hormones, their signaling, and the drug development of their analogues, especially those acting as dual and triagonists. Moreover, the main investigations into animal models and ongoing clinical trials using these unimolecular dual and triagonists are included which have demonstrated their safety, efficacy, and beneficial effects on the CV system. These therapeutic strategies could greatly impact the treatment of CVD with unprecedented benefits which will be revealed in the next years.

## 1. Current Challenges in the Treatment of Cardiometabolic Diseases

Population aging and the acquisition of sedentary lifestyle patterns in the population are increasing the prevalence of obesity and type 2 diabetes (T2DM). These two metabolic conditions are the leading causes of morbimortality worldwide and produce an important socioeconomic burden, mainly in the form of cardiovascular disease (CVD) [1]. The need to effectively reduce this major health problem has led to intensive research to find new therapies.

CVD and the acute events associated with this are the clinical manifestation of the atherosclerosis process, a chronic disease of the arteries which is sped up by the presence of metabolic alterations [2,3]. Currently, atherosclerosis is considered a metabolic and inflammatory disease that results from the interaction of circulating lipoproteins with cells of the innate and adaptive immunity system [4] and of the vascular vessel wall. The enhanced permeability and the activation of the endothelial layer induced by cellular dysfunction is one of the key mechanisms of disease initiation. Dysfunctional endothelium facilitates the adhesion, retention, and migration of immune cells and lipoproteins in the subendothelial space. These lipoproteins, mostly low-density lipoproteins (LDL), undergo different chemical modifications which are cleared by monocyte-derived macrophages forming lipid-loaded foam cells. These cells accumulate and constitute fatty streak incipient lesions. Along with macrophages, T and B lymphocytes are recruited into atheromas where they coordinate the inflammatory response by secreting mediators that exert divergent effects on lesion progression [5]. Proatherogenic T helper 1 (Th1) and Th17 cells are highly pro-inflammatory, and Th2 are anti-inflammatory and regulatory T (Treg) cells, which are a minor subpopulation of CD4+ T lymphocytes, suppress the activity of the effector Th cells [6]. Along with these processes, vascular smooth muscle cells (VSMC) transition from a quiescent and contractile toward a secretory, migratory, and proliferative phenotype [7]. VSMCs and the extracellular matrix below the endothelial layer form the fibrous cap that protects the increasing necrotic lipid core from ruptures [8] and constitute an asymptomatic stable plaque or fibroatheroma. Notwithstanding, the secretion of different death factors and the immune cell imbalance, such as a low Treg/Th17 cellular ratio [9], eventually promote the formation of critically unstable plaques. These plaques, characterized by big necrotic cores covered by thin fibrous caps are the hallmark of acute coronary ischemic syndromes which are caused when these plaques rupture [10].

The current therapeutic options to reduce acute events in advanced atheroma vulnerable plaques are very limited. Under intensive investigation, strategies were targeted to reduce the residual inflammatory risk associated with these plaques. Thus, the CANTOS trial that evaluated the anti-IL1β antibody did not get approval for CVD prevention despite a successful 15% reduction in CVD events [11]. On the other hand, the CIRT trial which examined methotrexate did not change cardiovascular (CV) events [12]. By contrast, the beneficial effect against CVD of the ancient colchicine has been demonstrated in LoDoCo, LoDoCo2, and CoLCoT clinical trials, thus offering a promising curative effect of long-term administration in cardiovascular patients [13].

Nevertheless, preventive measures to restore metabolic control and the reduction of risk factors such as hyperlipidemia, hypertension, insulin resistance (IR), and adipose tissue dysfunction are still the first line of treatment [1]. These preventive measures include bariatric surgery and lifestyle changes, as well as combinations of therapeutic drugs, addressed to specific metabolic targets. Although these are costly strategies, they have substantial beneficial effects on the incidence of CV-acute events and, therefore, are so far the best treatment to efficiently reduce the morbimortality and the socioeconomic burden of metabolic diseases [14].

Notably, many investigations have shown that bariatric surgery exerts important beneficial effects on T2DM resolution beyond the dietary restrictions caused by the surgery and the subsequent weight loss. These effects include increased insulin sensitivity, β cell activity, and decreased fat deposition among others that improve global metabolic health [15]. On the other hand, limited remission has been observed in patients with insulin dependence at the time of the surgery with total success in T2DM patients that had less than 8 years of disease onset. Despite this, improvements in CVD have been attributed to surgery in T2DM regardless of the use of insulin having protective actions against death produced by major CV events, including non-fatal acute myocardial infarction, stroke, and heart failure compared with patients that did not undergo surgery. Because of the improvement in the metabolic disease, whose mechanisms are complex and are not completely deciphered, these surgeries are called “metabolic surgeries” [16] and are a great promise in the prevention of CVD. However, the practice of bariatric surgery is expensive, invasive, and not sufficiently widespread to manage the obesity–T2DM pandemic.

One key underlying mechanism behind the success of metabolic surgeries in the remission of T2DM is the cooperative improved action of the gastrointestinal hormones. These hormones include peptide YY, oxyntomodulin (OXM), cholecystokinin (CCK), ghrelin, glucose-dependent insulinotropic polypeptide (GIP), glucagon-like peptide-1 (GLP-1), and glucagon. Therefore, these findings along with other investigations set the grounds for a new field in T2DM therapeutic approaches based on the use of the dual and triagonists of these hormones which all exert their functions through G protein-coupled receptors (GPCR).

In the next sections, a revision of the advances in therapies that combine different hormones for T2DM treatment is described. In general, these therapies have shown to significantly improve metabolic control beyond their effect or null effect as monoagonists and some of them have also been demonstrated to exert important CVD benefits.

## 2. The Biology of the Preproglucagon Derived HORMONES: Glucagon, GIP and GLP-1

### 2.1. The Biology of Glucagon

**Figure 1:**
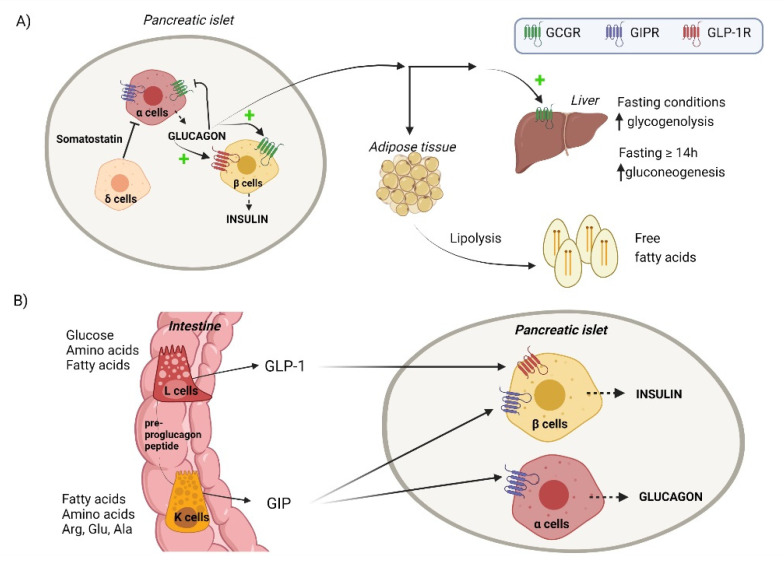
Main actions of glucagon, GLP-1, and GIP hormones. (**A**) In fasting conditions, α cells produce glucagon. In the liver, glucagon through GCGR activates the main regulated metabolic enzymes of glycogenolysis within the first hours of fasting and gluconeogenesis in fasting periods longer than 14 h. In β cells, glucagon also signals through GCGR and GLP-1R and stimulates insulin release which will promote the internalization of the hepatic glucose produced during fasting by periphery tissues. Glucagon production is inhibited by β and δ cell-derived factors like insulin and somatostatin, respectively, and by itself through a negative feedback loop. (**B**) The incretin hormones GLP-1 and GIP are secreted by the L and K cells of the intestine, respectively, in response to feeding and have insulinotropic actions through their respective receptors in β cells. While GLP-1 production is induced by glucose, fatty acids, proteins, and amino acids, the release of GIP is mostly induced by dietary fat and amino acids, especially arginine, glutamine, and alanine. GIP also displays a glucanotropic action through GIPR in α cells which explains the postprandial rise of glucagon after an amino acid-rich meal. Created with BioRender.com.

The glucagon hormone is a 29-amino acid hormone peptide and the main product of pancreatic α-cells. Like insulin, glucagon is synthetized as a precursor molecule, the preproglucagon peptide, which renders glucagon in α-cells by several proteolytic breaks [17]. Contrary to insulin, the preproglucagon peptide is processed to give rise to other two products, the incretin hormones GLP-1 and GIP, which are mainly produced in the intestine due to the presence of the proteases that generate these peptides (Figure 1).

The glucagon was discovered as a hyperglycemic pancreatic factor contaminating the insulin, hence its definition as a glucose agonist. The characterization of the hormone revealed that the hyperglycemic action was exerted through the glucagon receptor (GCGR)-mediated activation which enhanced glucose production via hepatic glycogenolysis and gluconeogenesis. Since then, glucagon has been viewed as a counterregulatory hormone of insulin in the liver and a hormone that is produced as a response to hypoglycemic and fasting conditions. However, increasing recent evidence has redefined the metabolic role of the peptide and spotted the hormone as an insulinotropic agent through its cross-talk with β cells and, therefore, questioning the dogma of glucagon as a hyperglycemic hormone only [17].

In this sense, glucagon secretion is augmented up to 2-fold by variation of glucose levels, but it is markedly increased up to 10-fold by the amino acids glutamine, arginine, and alanine, indicating that hypoglycemia is not the strongest inducer of glucagon secretion by α cells [17]. On the other hand, as the direct treatment of α cells with glucose increases the secretion of glucagon, the dogmatic in vivo inhibition of glucagon secretion in the presence of hyperglycemia seems to be an indirect mechanism. This is consistent with findings describing an inhibitory mechanism by some β-cell-derived factors, such as insulin, zinc, and γ-aminobutyric acid, which produce a paracrine inhibition of glucagon secretion by establishing a coordinated hormone β-to-α cell crosstalk. Moreover, δ-cell-derived somatostatin also inhibits glucagon secretion. Hence, the α cell’s tone and activity are set and modulated by both nutrient disposal and the islet cell microenvironmental factors produced in response to metabolic signals and nutrients.

In line with the above is the finding of increased secretion of glucagon in humans after a mixed meal, probably attributed to the induction by the abovementioned amino acids. In addition, contradicting the hypoglycemic dogma, a typical postprandial rise in glucagon in both T2DM patients and normal subjects is classically observed. Other evidences support that oral glucose administration promotes α-cell glucagon secretion by an enteroendocrine GIP-dependent mechanism which is better described in the next sections [17].

On the other hand, α cell tone and glucagon insulinotropic effects are quite relevant in β cell function. In fact, under metabolic stress, this insulinotropic effect of glucagon seems to be more important than its role as a hyperglycemic agent. Thus, contrary to what is expected as its classical role in metabolism, in hyperglycemia and metabolic stress, α cells undergo hyperplasia. Furthermore, the interruption of proglucagon production under stress conditions impairs nutrient stimulation of insulin secretion, indicating that a proper β cell function action requires α cell homeostasis. Consistent with a role of α cell tone and glucagon in β cell function is also the finding that T2DM patients and mice fed a high-fat diet display enhanced α cell mass and hyperglucagonemia as a mechanism to reduce metabolic stress through the enhanced insulinotropic action of glucagon in the dysfunctional β cell mass. Interestingly, the elevated glucagon production in metabolic stress has shown an increase in prohormone convertase (PC)1 in α cells which increases the paracrine production of GLP-1, which, through its GLP-1 receptor (GLP-1R) in β cells, improves insulin secretion [18].

Altogether, these new findings about the roles attributed to α cells and glucagon in islet cell functioning and maintenance have spotted glucagon as a potential peptide to be used in a coordinated treatment of deranged carbohydrate metabolism associated with obesity and T2DM.

### 2.2. The Biology of the Incretin Hormones GIP and GLP-1

The word incretin refers to the glucose-lowering action of intestinal factors [19]. The incretin effect was described as a specific gastrointestinal effect that stimulated insulin secretion from pancreatic β cells in a glucose-dependent manner upon nutrient ingestion, an effect that was not observed when glucose was administered intravenously. The characterization of these revealed the existence of the incretin hormones GLP-1 and GIP. GIP was the first incretin hormone described also called the gastric inhibitory peptide due to its ability to inhibit gastric acid secretion [19]. The most well-documented action of incretins is the stimulation of insulin secretion in β cells through the activation of their receptors, the GIP receptor (GIPR) and GLP-1R. However, many other actions have been reported for incretins, such as the regulation of gastric motility, nutrient absorption, blood flow, and food intake [19]. Many other actions have been reported in different tissues and cell types due to the wide distribution of their receptors.

As mentioned above, GLP-1 is a peptide derived from the proglucagon precursor that results from the proteolytic cleavage produced by the PC1/3 in the intestine, but it can also be produced in the pancreas and in the hindbrain [20,21]. The PC1/3 action renders two forms: GLP-1(7–37) and GLP-1-(7–36)NH(2). In the intestine, GLP-1 is produced and secreted in a glucose-dependent manner by the enteroendocrine L cells which are mostly found in the distal intestine, the ileum, and the colon.

Likewise, GIP is a preproglucagon peptide produced in the enteroendocrine K cells of the upper intestine that results from the posttranslational cleavage of the preprohormone proGIP. The most prevalent circulating form of GIP incretin is the GIP(1-42) produced by the action of the PC1/3, while the shorter form, GIP(1–30NH2), is the result of the PC2 in the intestine and pancreatic α cells. Both GIP peptide forms have insulinotropic activity in β cells, but this activity is less pronounced than that observed for GLP-1. In the intestine, the production of GLP-1 is promoted by different sugars such as glucose and galactose as well as fatty acids, proteins, and amino acids, while the release of GIP is mostly induced by dietary fat.

Fasting and postprandial GIP levels are higher than those of GLP-1 and seem to be highly important in healthy subjects. Interestingly, GIP levels are also raised in fasting conditions which is consistent with its function of exerting a glucanotropic activity in a glucose-dependent manner and having the highest activity in hypoglycemic conditions.

Both GIPR and GLP-1R in β cells are strongly affected by metabolic stress and display a reduced expression and activity. However, GIPR is more sensitive to this stress which suggests that its impaired activity could be behind the homeostatic metabolic loss under stress conditions [19]. In this regard, studies in T2DM have demonstrated impaired GIP insulinotropic action due to low activity of β cells as well as an important glucagonotropic activity in α cells, even in hyperglycemia, that characterizes T2DM as demonstrating a dysfunctional GIPR function in α cells [22]. On the other hand, mild T2DM patients also display resistance to exogenous-infused GIP peptide for its insulinotropic action, demonstrating dysfunctional GIPR-dependent signaling [23]. Notably, as described above, the glucanotropic action of GIP in α cells seems to be promoted by postprandial amino acids [24].

Hence, the above investigations have pointed to GIP as an important factor mediating the incretin effect and the glucose tolerance by dual actions on α and β cells. Nevertheless, the preclinical studies with therapies based on GIP rendered conflicting results and were soon discarded as potential human therapies.

Because of the complex biology of GIP, the development of GLP-1 analogues centered the attention for drug development on the treatment of T2DM. GLP-1-based therapies have had great success in the last decade in both the preclinical and clinical stages [25,26]. In fact, most of the GLP-1 analogues have been demonstrated to delay CVD by exerting pleiotropic and anti-inflammatory effects in the CV system and currently are in the first line for T2DM treatment and the prevention of its complications.

Both GLP-1 and GIP are cleared rapidly by renal elimination and are susceptible to degradation by the dipeptidyl peptidase 4 (DPP4), a membrane-bound serine peptidase mostly expressed in the liver, intestine, and kidney, although it is also found in plasma in a soluble form [19]. DPP4 is the primary enzyme that inactivates both incretin hormones but is less active against GIP. Notably, soluble DPP4 correlates with insulin resistance and adipose tissue inflammation [27]. Therefore, DPP4 inhibitors for the treatment of T2DM also had great success, being highly effective through mechanisms involving not only an augmented half-life of incretins but also by additional mechanisms [28]. These mechanisms included improved anti-hyperglycaemic and glucagonotropic action by increasing GIP stability.

Because of the new knowledge of the importance and complexity of GIP biology in α and β cell tones, and because DPP4 inhibition had additional beneficial effects compared to GLP-1R monoagonism, GIP has been reconsidered for the development of dual GLP-1/GIP agonists in the treatment of obesity and T2DM.

### 2.3. Metabolic Effects of the GPCR Dependent Signaling

GCGR, GIPR, and GLP-1R are members of the class B GPCR superfamily which are characterized by being highly structurally similar. All three receptors are membrane heterotrimers. Each monomer has a characteristic structure with seven transmembrane alpha helices. Ligand binding to the receptor induces a conformational change that engages the signal transduction through the membrane and the activation of a G protein complex which releases a Gα subunit.

The most known effect of the GCGR-dependent signaling is the hepatic production of glucose via the activation of glycogenolysis at the initial phases of fasting and gluconeogenesis after prolonged (around 14) hours of fasting by modulating the main metabolic enzymes of these metabolic pathways. Likewise, in adipose tissue, GCGR transduction promotes the release of fatty acids to increase nutrient disposal [29]. GCGR is also expressed at similar levels in β cells where, as mentioned above, it has insulinotropic action through paracrine α-to-β cell crosstalk. Interestingly, the main insulinotropic activity of glucagon in β cells seems to be through binding to the GLP-1R, although this activity is lower than that of GLP-1/GLP-1R signaling. GCGR is also found in α cells to control glucagon secretion through a feedback loop to control bioactive glucagon, as mice deficient in GCGR display enhanced glucagon levels. On the other hand, in vivo blockade of GCGR-signaling with highly specific antibodies effectively reduces blood glucose and improves glucose tolerance in obese and lean mice, as well as in monkeys [30].

Notwithstanding, GCGR has a wider tissue distribution including the brain, heart, kidney, adipose tissue, and gastrointestinal tract. Thus, glucagon regulates, through neural signaling, energy expenditure, and satiety [18].

The most important metabolic effect of incretins is in the pancreas. GIP and GLP-1 bind to their respective GPCR in β cells and transduce the production of insulin secretion. GPCR activation leads to Gα-mediated stimulation of adenylate cyclase for the production of cAMP which, through allosterism, activates the protein kinase A (PKA) and the exchange protein directly activated by cAMP 2 (Epac2), increasing intracellular calcium and the release of insulin from its vesicles. 

GLP-1R is coupled to the cognate Gsα of the hormone GPCR but also with two other G trimers subunits, Giα, and Gqα, as well as with β-arrestin-1 [19,31]. Consistent with this, the knockdown of β-arrestin-1 in rat insulinoma cells decreases the ability of GLP-1 to promote glucose-stimulated insulin secretion. In addition, GLP-1R-mediated signaling promotes the survival of β cell mass by increasing proliferation and inhibition through apoptosis. Specifically, GLP-1R inhibition of apoptosis cell death is mediated by both PKA-phosphoinositide 3-kinase/protein kinase B (PI3K/AKT) via the epidermal growth factor receptor (EGFR) and by GLP-1R/PKA-PI3K-mitogen-activated kinases ERK pathways. GLP-1/GLP1-R promotes the preservation of β cell mass by increasing the transcriptional factor pancreatic duodenal homeobox 1 (PDX1) via the PKA/cAMP/response element-binding protein (CREB) pathway. In addition, GLP-1 signaling reduces endoplasmic reticulum (ER) stress by PKB/Akt and CREB activation. The relevance of GLP-1R in β cells is demonstrated in *Gipr-/-* mice, who display impaired glucose tolerance and defective glucose-stimulated insulin secretion.

GLP-1R is also expressed in α cells, a highly relevant clinical finding due to the GLP-1 inhibitory effect on glucagon secretion, which has a major effect on circulating glucagon. GLP-1R has also been found in the kidney, lung, gastric mucosa, heart, hypothalamus, hippocampus, and immune cells, through which they exert important CV benefits which are fully explained elsewhere [19,26]. GLP-1 also stimulates somatostatin from the δ cells that express GLP-1R. 

Like GLP-1R, the activation of GIPR signaling transduces coupled Gαs to raise cAMP levels and intracellular calcium through PKA and Epac2 pathways for insulin-containing vesicle exocytosis. In addition, GIPR signaling also activates different kinase cascades including PI3K, PKB, MAPK, and phospholipase A2. Protection from β cell apoptosis has also been observed for the GIP/GIPR pathway through CREB activation and the inhibition of the stress MAPK p38 and c-Jun N-terminal kinase (JNK)MAPK. Thus, specific deletion of *Gipr* in β cells worsens streptozotozin-induced β cell destruction while GIP infusion reduces apoptosis in diabetic mice. GIP/GIPR also promotes insulin gene transcription and biosynthesis [32].

As mentioned above, an important function of GIP is its glucagonotropic action by signaling through GIPR in α cells [22]. Similar to GPCR in β cells, GIPR-signaling in α cells leads to an increase in cAMP levels and the activation of PKA signaling, and increased calcium to promote glucagon secretion. Unlike GLP-1, GIP induces a major response to mixed meals and macronutrients. First, GIP potentiates α cell glucagon production which facilitates α to β cell communication; secondly, it adds to the GLP-1R/GCGR signaling effect in β cells; and third, it exerts a direct insulinotropic action on β cells through its receptor. Besides β and α cells, GIPR is also expressed in δ and γ cells [33]. In fact, increasing studies are showing a complex β, α, and δ islet communication which seems to be partly coordinated by GIP/GIPR signaling. In this manner, GIP signaling in pancreatic islets leads to a more potent insulinotropic response and potentiates α-to-β cell crosstalk. 

GIPR is also expressed in the gastrointestinal tract, adipose tissue, heart, testis, bone, lung, pituitary, adrenal cortex, and multiple regions of the central nervous system [34].

Many genetic and experimental studies pointed to the GIPR-signaling antagonism as a logical therapeutic strategy against diet-induced obesity [33]. However, both antagonism and agonism of the GIPR prevented weight gain in preclinical models [33]. A logical explanation has been given consisting of desensitization of the receptor by a long-term sustained GIPR agonism that mimicked the functional GIPR antagonism, thus promoting a negative energy balance and reducing body weight [35]. Moreover, recent preclinical investigations described below suggest that, in combination with GLP-1, GIP/GIPR-signaling could have potential additional clinical benefits in humans.

Because of the above, naturally occurring dual-agonists have also been spotted as potential therapeutic targets for the treatment of obesity and associated metabolic derangement. One of these is the OXM peptide which is a dual GLP-1R/GCGR agonist. OXM is a gut-derived hormone peptide that, despite having a lower affinity toward both GLP-1R/GCGR than the cognate ligands, reduces body weight by increasing energy expenditure [36]. Thus, in humans and in experimental models, it has been shown to reduce body weight loss and food intake, increase energy expenditure, and amplify glucose-induced insulin secretion. Given that no specific OXM receptors have yet been identified and because of a lack of the OXM effect in *Glp-1r-/-* mice, its insulinotropic action has been attributed to GLP-1R signaling.

## 3. Development of GPCR Agonists

### 3.1. GLP-1R and GIPR Agonists

As mentioned above, a wide range of structurally and chemically optimized GLP-1 analogues have been formulated and are currently in clinical practice for the treatment of obesity and T2DM [21,37,38]. The complex pharmacokinetic profile of the native GLP-1 peptide, with a half-life of 2–3 min in plasma [20,39], is the main limiting factor for human GLP-1 clinical use. Therefore, the developed GLP-1R agonists are human GLP-1 hormone derivatives with modifications aimed at increasing molecular stability by preventing DPP4 degradation and activity, including aminoisobutyric acid (Aib) replacement. Some of the GLP-1 analogs are derived peptides of the exendin-4 substance, a natural GLP-1 agonist obtained from the saliva of the lizard species *Heloderma suspectum*, as detailed by us in [26]. Regarding its application for pathologies other than T2DM, as mentioned before, numerous studies have demonstrated CV benefits for almost all GLP-1 agonists [40,41] which is due to the wide expression distribution of its GLP-1R.

One of the problems of GLP-1 agonist therapy is the daily subcutaneous injection as a route of administration. Therefore, developments of the initial molecules have been to diminish the frequency of administration. Among GLP-1R agonists, the small acetylated peptide semaglutide is notable for having an oral formulation, approved in 2019 [42], which was first prescribed as a once-weekly subcutaneous injection. Semaglutide is a human GLP-1 analogue derived from the GLP-1R agonist liraglutide, modified by attaching long chain fatty acids to increase albumin affinity and resistance to DPP4 degradation [43,44]. Due to its low molecular weight and long half-life [43], it was considered the ideal GLP-1 agonist candidate for oral delivery. The oral form of semaglutide was developed by co-formulating semaglutide peptide with permeation enhancer sodium N-(8-[2-hydroxybenzoyl]amino)caprylate (SNAC), which allows for absorption and protection from the degradation of semaglutide in the stomach [45].

Unlike GLP-1, GIP has attracted little interest in the pharmaceutical area due to the conflicting results mentioned in the previous section as well as the lack of understanding of GIP dose-response actions [46]. In fact, there are no clinical therapies using GIPR agonists in monotherapy, and the role of incretin GIP in the CV system is still not well understood. However, the results of GLP-1R/GIPR dual agonists have increased the interest in this incretin. On the other hand, the pharmacological combination of GLP-1 with glucagon, GIP, and the other hormones structurally related in an intermixed-unimolecular form reflects the important evolution that the hormone-derived GPCR agonists-based therapies have undergone in the fields of metabolism and CVD [21,47,48].

### 3.2. GLP-1R and GCGR Dual Agonists

Based on the naturally occurring dual agonists, the preclinical results, and human data for OXM, the combined stimulation of GLP-1R/GCGR stands out as an important therapeutic strategy for blood glucose control and decreases in body weight [49]. Moreover, emerging evidence points to the potential of this therapeutical option to treat not only T2DM and obesity but also related comorbidities [49,50,51].

Most of the dual GLP-1R/GCGR agonists are unimolecular peptides that activate downstream effectors of both receptors and were discovered by rational design. One of the most decisive aspects of the efficacy and functionality of dual agonists is the relative activation of each receptor since overactivation of either of them can lead to adverse effects [52]. The general approach to generating GLP-1R/GCGR dual agonists is to use glucagon, given its affinity to GLP-1R or OXM as the scaffold to which amino acids specific to human GLP-1 or exendin-4, especially from the C-terminus, are introduced to increase GLP-1R activation. Exendin-4 backbone is also used in other strategies, including glucagon/OXM modifications in N and C-terminal, respectively, to gain potency [25]. To increase the stability of the unimolecular peptides, charged residues with helical-promoting properties can be introduced, such as Aib. Delivery can be improved through fatty acid acylation of the synthetic co-agonists for improved plasma protein binding [53,54]. 

Among the different developed drugs, SAR425899 is one of the most relevant. It is a unimolecular dual GLP-1R/GCGR agonist whose phase 1b clinical trial results were presented recently [52]. It is a hybrid peptide with a potent ability to activate both receptors, designed by including structural elements of glucagon in exendin-4, to include GCGR agonism whilst maintaining a potent GLP-1R activation. To achieve greater metabolic stability, other amino acid modifications and unnatural residues have been introduced [50].

Another dual GLP-1R/GCGR agonist is LY3305677, also known as mazdutide (R&D code: IBI362). Mazdutide is a long-acting single-chain synthetic peptide analogous to the mammalian OXM, modified by acylation to increase its half-life [55]. A similar dual GLP-1R/GCGR agonist is MEDI0382, also known as cotadutite, which is under development for T2DM comorbidities, nonalcoholic steatohepatitis (NASH), and chronic kidney disease [56]. MEDI0382 is a chemically synthetic linear peptide based on the human OXM and glucagon peptide backbone with a palmitic acid side chain [57] to facilitate binding to albumin and to increase the half-life [58]. Aminoacidic modifications of cotadutide to reduce proteolysis have been performed, such as a replacement of glutamine residues 20 and 24 with other amino acids not susceptible to deamination and the substitution of arginine 17 by glutamate [58].

### 3.3. GLP1R and GIPR Receptor Dual Agonists

With the aim of broadening the range of action for GLP-1R analogues, the first choice of comorbidities associated with T2DM [59], the combination GIPR analogues, has been developed in recent years [37]. In this regard, unimolecular GLP-1R/GIPR dual agonists, called “twincretins”, have shown synergistic effects and increased effectiveness and may even exert a favorable impact on the CV system [60]. These incretins seem to work in tandem; when plasma glucose levels are high, GLP-1 diminishes glucagon secretion while GIP reduces glucose concentration [61].

To generate GLP-1R/GIPR co-agonists, a chimeric sequence of human GLP-1 and exendin-4 is used as a scaffold and then different GIP residues, especially isoleucine 12 and lysine 6, to achieve GIPR potent activation, are introduced [62]. Similar to GLP-1 and glucagon dual analogues, peptide modifications by acylation and polyethylene glycol(PEG)ylation increase stability which allows a reduction in dose frequency [25].

Among the different dual GLP-1/GIPR agonists, based on the results of their respective clinical trials, the most important are two unimolecular twincretins [63]: tirzepatide once weekly (previously LY3298176) [64] and NN0090-2746 once daily (previously known as RG7697 and MAR709) [65].

Tirzepatide is a synthetic peptide developed by Eli Lilly (Indianapolis, IN, USA). This twincretin is based on the human GIP sequence, with modifications in the peptide backbone to obtain dual receptor-activating activity and prolonged half-life [20]. It is a linear molecule of 39 amino acids that includes residues from GLP-1 and 2 non-coding amino acids [66]. They are two Aibs at positions 2 and 13, which are responsible for their long half-life, approximately 5–6 days, due to their high affinity for albumin and the prevention of peptidase degradation [67]. In addition, fatty-acylation of the lysine 20 residue with a C20 diacid, linked via hydrophilic linkers (γ-Glu-2xAdo, gamma glutamate, and bis-aminodiethoxyacetyl), allows for covalent binding with albumin [68]. Moreover, the C-terminal of the peptide is elongated with the C-terminal domain of exenatide [66]. Currently, this co-agonist is synthesized using a hybrid solid-phase peptide synthesis/liquid-phase peptide synthesis (SPPS/LPPS) approach, achieving high purity and yield [69].

The other main twincretin is NN0090-2746, a fatty-acylated GLP-1R/GIPR dual agonist [65]. It is a 40-amino acid synthetic peptide containing an α-Aib residue at position 2, to prevent DPP4 degradation [70,71]. The sequence is a homologue of both incretins with acetylation at the C-terminal lysine with a saturated C16 lipid to improve its pharmacokinetic profile and stability [71].

Due to the structural similarities between GLP-1 and GIP, there are several other unimolecular synthetic peptides with balanced agonism at both the GLP-1R and GIPR, with metabolic benefits in different animal models. In general terms, these co-agonists present different rational designed modifications within the glucagon-based core sequence. The changes include PEGylation and acetylation to reduce the dose frequency, the introduction of GLP-1 and GIP-specific residues, and amino acid substitutions to protect against DPP4 cleavage [62].

### 3.4. Other-Dual Agonists Peptides Based on GLP-1

Other peptide/peptide multiagonists targeted to multiple pathways have been developed with demonstrated metabolic benefits. They include the combination of the GLP-1 with other related hormones, such as the anorexigenic CCK, gastrin, or xenin, by generating dual agonists of their respective receptors [72]. However, these new emergent dual agonists merit a separate revision.

### 3.5. GPCRs Triagonists

The clinically effective monoagonists and the success of the preclinical and clinical results of dual agonist peptides discussed above have boosted the development of triple agonists in the GPCR system. In fact, various unimolecular multiagonists are in preclinical and clinical trials as they achieved sustained metabolic improvements by the simultaneous activation of multiple hormone receptors [72]. Of particular relevance are the GLP-1R/GIPR/GCGR triagonists, developed on the rationale of a high degree of homology of the three endogenous peptides, as well as the strong cross-affinity of their receptors. Potentially, they could combine three synergic approaches: enhancement of insulin secretion by GLP-1R agonism, weight loss mechanisms of GCGR, and beneficial effects of glucagon on energy expenditure, lipid and glucose metabolism, and the metabolic homeostatic role, insulinotropic and glucagonotropic, of GIP in islet cells [25,63]. Thus, the development of unimolecular multireceptor agonists is seen as a future strategy to treat metabolic and CVD with unprecedented efficacy.

The unimolecular GLP-1/GIP/glucagon peptides have been designed using the scaffold of the precursor hormones in which specific residues from the other peptides are introduced to achieve a mixed agonism resulting in unimolecular synthetic peptides with intermixed affinity [25]. Exendin-4, glucagon, and OXM are the most frequently used scaffolds to develop GLP-1/GIP/glucagon peptide analogues. The developed unimolecular GLP-1R/GIPR/GCGR triagonists are similar in length to endogenous glucagon peptides with sequence homology with exendin-4 or human GLP-1 [73]. Other triagonists used the GLP-1R/GCGR dual agonist, and an insertion of a GIP-specific amino acid to yield potent triagonisms [74]. Other approaches include the development of a hybrid GIP-OXM peptide by replacing residues from the N-terminal of OXM with D-Ala-GIP (DA^2^GIP-OXM) [75] or triagonists derived from the three endogenous hormone peptide sequences ([DAla^2^]GLP-1/glucagon) [25]. 

Three of the different triagonists, MAR423, HM1521, and SAR441255, are under clinical development. First published by Finan et al. 2017, MAR423 is a highly effective triagonist of GLP-1R/GIPR/GCGR developed by Novo Nordisk by iterative residue changes and refinement of the chemical structure, with amalgamated amino acids derived from each of the native hormone sequences [74]. In initial clinical trials, the peptide has been shown to be resistant to DPP4 cleavage due to the addition of Aib in position 2. It has high solubility and a long-lasting effect thanks to the introduction of an acyl moiety and has a C-terminal-extended domain derived from exendin-4 [25].

HM1521 is a once-weekly administered long-acting triagonist described by Hanmi Pharmaceuticals. The peptide is based on the glucagon sequence and is conjugated via the non-peptidyl flexible linker to the human aglycosylate Fc fragment [72].

The third GLP-1R/GIPR/GCGR triagonist is SAR441255, and the results of its phase 1 study have just been published [76]. It is a synthetic peptide based on the exendin-4 sequence, with amino acids of GLP-1 at positions 17 and 21, glucagon at position 18, and of GIP at positions 19 and 28, which enhances GCGR and GIPR activation. In addition, to potentiate its half-life, an Aib acid residue is introduced in position 2 which prevents DPP4 cleavage. To increase its delivery and transport, a palmitic acid side chain is included at the lysine residue, which facilitates albumin binding [76].

Although GLP-1R/GIPR/GCGR triagonists are the most prominent in terms of current development, there are also triagonists based on other GPCR. One of them is the *Xenopus* (x) GLP-1/GCG/CCK2 receptors triagonist (also called xGLP/GCG/gastrin) which is addressed against GLP-1, GCG, CCK1, and CCK2 receptors. It is a potent and long-acting triagonist based on the structures of xGLP/GCG-15 and gastrin-6 with a fatty acid side chain coupled to the amino group of Lys, synthesized using SPSS. The results in mouse models have validated its therapeutic potential for the treatment of obesity and diabetes [77].

## 4. Studies of GPCR Agonists in Mouse Models

Extensive research in mouse models has provided extensive knowledge about the potent and efficient effects of GLP-1 analogues in T2DM, obesity, and their comorbidities which have been described in recent excellent reviews [21,37] and meta-analyses [59]. To avoid duplicities, in this section, a brief description of the studies performed in diabetic and CVD mouse models with the most recently developed GPCR agonists is presented.

### 4.1. GPCR Agonists in Monotherapy: Semaglutide and GIPR Agonist Studies in Mouse Models

As mentioned before, the most recent studies of GLP-1 analogues were focused on reaching a long-lasting effect and a change in the administration route. These two conditions were reached with the development of semaglutide which, on top of improving metabolic control, has been shown to display CV protection in animal models. 

In this sense, Western diet-fed *Ldlr* deficient (*Ldlr-/-)* and *Apoe-/-* mice treated with semaglutide successfully prevented plaque lesion development and body weight gain, independently of glucose-lowering. Semaglutide also diminished the plasma inflammatory markers in a lipopolysaccharide-induced inflammatory model in mice. Interestingly, the peptide partially reverted in mouse aortic atheromas, the over-expression of genes involved in lipid metabolism and signaling, leukocyte recruitment, rolling, and extravasation, as well as extracellular matrix protein turnover and plaque hemorrhage [78]. The atheroprotection exerted by semaglutide was not mediated by endothelial or hematopoietic cells, since similar results were obtained in mice regardless of the presence of GLP-1R expression in these cell types.

Contrary to GLP-1 analogues, the studies in mouse models of T2DM treated with GIP analogues used in monotherapy did not reach the clinical phase because of the conflicting results in body weight, glucose disposal, and energy expenditure. Regarding the atherosclerosis effects of GIP in monotherapy, studies have also pointed to both the anti-atherogenic and pro-atherogenic effects of GIPR agonists [79]. Because of these conflicting results, no GIPR agonist is currently being clinically used in monotherapy [26].

In rodent studies, GIPR-null mice showed inferior body weight and fat mass than *wild-type* mice after a high-fat diet, due to increased energy expenditure and lipogenesis [80]. Despite these results, GIPR agonists and acyl-GIP administration also decreased body weight caused by reduced food intake in a dose-dependent and central nervous system (CNS)-mediated manner [81,82]. Chronically GIP-overexpressing transgenic mice showed similar effects, suggesting the protection of the pathway against obesity. These beneficial effects consisted of a concomitant improved glucose homeostasis and insulin sensitivity as well as the down-regulation gene expression of lipid metabolism and inflammatory signalling pathways [83]. These results reporting similar actions by antagonism and agonism of GIPR were later explained by other authors which reported desensitization of the receptor by a long-term sustained GIPR agonism that mimicked the functional GIPR antagonism, thus promoting a negative energy balance and reduced body weight [35].

In mouse models of atherosclerosis, GIP produced relevant changes in atheroma lesions. Experimentally increased GIP in *Apoe-/-* and *db/db* diabetic mice reduced atherosclerotic plaque lesions and foam cell formation by glucose-independent mechanisms [34,84,85], indicating atheroprotection. Likewise, the administration of a GIP active form into *Apoe-/-* mice increased plaque stability with enhanced collagen content [86] and decreased metalloproteinase activity. GIP also was shown to impair parameters for leukocyte adhesion in endothelial cells and migration of VSMCs which are associated with atheroma plaque development [34,85].

Another reported effect of GIP administration in *Apoe-/-* mice has been a reduction in in vivo and ex vivo foam cell formation by the downregulation of CD36 and acyl-coenzyme A:cholesterol acyltransferase-1 (ACAT-1) in macrophages and cAMP activation. The same results were observed in human U937 macrophages, which also reduced oxLDL uptake and cyclin-dependent kinase 5 (*Cdk5*) expression when treated with a GIP analogue [87]. The infusion of an inactive form of GIP, GIP(3–42), had no effect on these parameters or on atherosclerosis [85].

In general terms, the preclinical investigations which are better revised elsewhere [33] raised safety issues to further exploring GIP analogues in monotherapy. However, the complex beneficial role of the hormone in metabolism and its potential in other tissues hold the hope to be used in combination with the GLP-1 analogues.

### 4.2. Studies in Mouse Models of Dual Agonists of GPCR 

As described in Section 3, dual GCPR agonists are based on peptides that activate two of the three glucagon-derived hormones, being one of the two activations of GLP-1R. These dual agonists were proven to have beneficial effects in preclinical studies, some of which are described below.

#### 4.2.1. Effects of Dual GLP-1R/GCGR Agonists 

The dual GLP-1R/GCGR agonists best characterized are three: SAR425899, mazdutide, which is called also LY3305677, and cotadutide, also known as MEDI0382.

Treatment of high-fat diet-fed and diet-induced obese (DIO) mice with the dual agonist that share pharmacological properties with SAR425899 reduced body weight and fat mass compared to mice treated with the GLP-1R agonist liraglutide. Differences were attributed not only to reduced food intake but also to increased total energy expenditure by using fat and carbohydrate in oxidative processes [50,88]. In the same investigation, the monkey-specific dual agonist reduced total energy intake to 60–70% during chronic treatment of DIO non-human primates [50]. On the other hand, SAR425899 significantly enhanced plasma glucose in overnight fed *Glp-1r-/-* mice due to the activity of GCGR but not of GLP-1R [88]. Although, in diabetic *db/db* mice, both SAR425899 and liraglutide agonists showed similar effects on glycemic control evaluated as a decrease in the glycosylated hemoglobin, HbA1c, levels [50].

The GLP-1R/GCGR dual agonist LY3305677, also known as mazdutide, and IBI362 decreased body weight in both *Gcgr-/-* and *Glp1r-/-* mice, hence demonstrating dual agonism. In other research, LY3305677 significantly decreased food intake and improved glucose tolerance and insulin secretion in a dose-dependent manner in both DIO and streptozotocin-induced diabetic mice [89]. Moreover, fibroblast growth factor 21 (FGF21) levels were increased, a factor that reduces glucose levels and enhances fat expenditure, in dual agonist-treated mice. Similarly, improved lipid metabolism parameters were observed which consisted of improved hepatic lipid delivery shown by a decrease in serum triglycerides and in the levels of serum proprotein convertase subtilisin/kexin type 9 (PCSK9) which results in enhanced clearance of LDL from the blood. Interestingly, LY3305677 also increased fatty acid oxidation along with reduced liver triglyceride content and increased serum ketones, a parameter that indicates extensive use of fat which could be responsible for weight loss.

The third GLP-1R/GCGR dual agonist MEDI0382, also called cotadutide, improves glucose homeostasis and reduces body weight and food intake in rodents and non-human primates. In DIO mice, it produces a higher weight loss compared with that observed in DIO mice treated with the GLP-1R agonist liraglutide. The differences were attributed to augmented energy expenditure mediated by the GCGR agonism, while decreased food intake was proposed to be mediated mostly through GLP-1R signaling [58]. Importantly, MEDI0382 reduced the hepatic burden and NASH through a reduction in hepatic lipid content, as well as by an improved mitochondrial function. MEDI0382 was proved to be more effective at reducing hepatic fibrosis than liraglutide despite similar weight loss in two independent mouse models of NASH [90].

#### 4.2.2. Studies with GLP-1R/GIPR Dual Agonists 

As mentioned in Section 2, better knowledge of GIPR biology and observations made in clinical studies using DPP4 inhibitors pointed to additional potential benefits in the use of GLP-1R/GIPR co-agonists. On the other hand, preclinical experiments in *wild-type* and DIO mice with the disruption of one of the two incretin systems with specific antibodies also underlined a coordinated response of the two incretin-dependent pathways. Hence, the dual GLP-1R/GIPR agonism has a potent insulinotropic action, while GIPR responses have a compensatory glucagonotropic effect and a key role in islet cell tone maintenance. In general terms, experimental research has provided evidence for the additional benefits and some of the mechanisms behind the potentiation of dual agonism [91]. However, CV beneficial effects remain to be investigated.

The GLP-1R/GIPR dual agonists that are better characterized are tirzepatide, also known as LY3298176, NN0090-2746, previously known as RG7697, and MAR709.

The dual agonist tirzepatide induces glucose-dependent insulin secretion in vitro and in vivo. In high-fat-fed obese mice, tirzepatide improved glucose homeostasis and insulin sensitivity in musculoskeletal muscle and white adipose tissue, while it decreased body weight [92]. In cell lines, LY3298176 activated both GIPR and GLP-1R signaling and, in DIO mice, showed a glucose-dependent insulin secretion action and improved glucose tolerance. In DIO mice, LY3298176 potently decreased body weight, through augmented fatty acid oxidation, and food intake in a dose-dependent manner with a greater effect than the GLP-1R agonism alone [68]. These observations are consistent with the upregulation of genes associated with glucose, lipid metabolism, and branch amino acids catabolism in brown adipose tissue in high-fat diet-fed obese mice [92]. 

In DIO mice, GLP-1R/GIPR co-agonist NN0090-2746 also decreased body weight, food intake, fat mass, and cholesterol levels. In addition, the dual agonist also lowered insulin secretion and improved glucose tolerance in DIO mice, *db/db* mice, and Zucker diabetic fatty rats. In monkeys, the treatment with NN0090-2746 during a glucose infusion assay increased insulin secretion and decreased blood glucose more effectively than liraglutide [62]. In another investigation conducted with DIO mice, NN0090-2746 improved metabolic syndrome features and reduced plasma biomarkers of systemic inflammation and CV risk [91].

### 4.3. Studies in Mouse Models with the GLP-1R/GIPR/GCGR Triagonists

Some of the most recent drug developments for T2DM and obesity are the triagonists for GLP-1R, GIPR, and GCGR (GLP-1R/GIPR/GCGR). Three drugs have been developed which are MAR432, SAR441255, and HM15211. MAR423 has been studied in DIO female and male mice and has shown to reduce hypercholesterolemia and body weight without excessive fat mass loss in both sexes, with a more pronounced decrease in steatohepatitis in female mice and an enhanced glucose tolerance in male mice [93]. In another study, which included mice deficient for each of the triagonist receptors, *db/db* and streptozotocin-treated mice as well as DIO and ZDF rats, MAR423 exerted a synergistic effect in which glucagon was more responsible for the increased energy expenditure. Moreover, the GIP agonism balanced the glucagon diabetogenic effect and potentiated the incretin effect of GLP-1, which mainly reduced caloric intake and improved glucose control [74].

SAR441255, another GLP-1R/GIPR/GCG triagonist, significantly reduced body weight via increased energy expenditure and non-fasting glucose levels in a DIO female mice model compared with the GLP-1R/GCG dual agonism. A similar experiment was reproduced in DIO and diabetic cynomolgus monkeys with no changes in body weight or in HbAc1 plasma levels between monkeys treated with SAR441255 or treated with a GLP1R/GCG dual agonist; although, fasting plasma glucose was significantly lowered in SAR441255 treated animals. Furthermore, SAR441255 was proven to be safe regarding glucose control in both DIO and diabetic cynomolgus monkeys and did not cause any major CV events in lean cynomolgus monkeys [76], which was highly relevant for further clinical development.

The third GLP-1R/GIPR/GCG triagonist, HM15211, reduced glycemia and body weight by increasing energy expenditure more effectively than once-daily liraglutide treatment in the mouse model fed with a methionine choline-deficient diet (MCD) [72,94].

Other combinations of triagonists are currently under development, although none of them have yet been studied in the context of their vascular effects. A new GLP-1R/GIPR/GCGR triagonist is XFL6, which restores glucose homeostasis and decreases HbAc1 levels, body weight, food intake, IL-6, and TNFα levels, compared with semaglutide treatment. In addition, it also ameliorated lipid metabolism and diabetic nephropathy parameters [95].

One last GLP-1R/GIPR/GCGR is DA2GIP-OXM, which in a high-fat-fed mice model reduced plasma glucose levels and increased secretion of insulin [96] without altering insulin resistance or food intake when administered acutely, 4 h before a glucose load, or once-daily [75].

Another change observed in the treatment of the animal models with the triagonists was a coordinated effect on islet cells. In streptozotozin (STZ)-induced diabetic mice, the twice-daily administration of (D-Ala2) GIP and (D-Ser2)-OXM[Lys38PAL] restored pancreatic morphology, increased insulin content, and improved β cell survival. Notably, cellular tracing experiments have also revealed that GIP and OXM analogues induce α-to-β cell transdifferentiation in a diabetic mouse model with β cell loss induced by streptozotozin [97]. Thus, this drug also holds promise for restoring β cell loss in T2DM or in Type 1 DM. 

Regarding the triagonists based on the activation of GLP-1R, GCGR, and CCK1 and CCK2, the triagonist Xenopus (x) GLP-1R/GCGR/decreased body weight in DIO mice to a greater extent than ZP3022, a dual GLP-1R/CCK-2R agonist, and liraglutide. xGLP1/GCG/gastrin treatment also increased the islet number, insulin secretion, and glucose control and tolerance in *db/db* mice better than ZP3022, cotadutide, or xGLP/GCG-15 [77].

## 5. Clinical Trials Using Class B GPCR Agonists

### 5.1. Recent Advances in Incretin Hormone Receptor Monoagonists in Humans

As described earlier, the GLP-1R agonists are in the first line of treatment for T2DM and obesity. Notably, all developed GLP-1R agonist drugs produce generally positive results in decreasing plasma glucose levels, lowering the Hb1Ac concentrations, and improving surrogate cardiometabolic markers, hence proving their effectivity not only in restoring carbohydrate metabolism but also in preventing death from CVD causes whose clinical trials are excellently reviewed elsewhere [98,99]. Specifically, beyond the metabolic effect on diabetic and/or obese subjects, [99] a meta-analysis carried out with eight different trials in T2DM patients, also reported the effect of different GLP-1R agonists on cardiovascular and kidney outcomes. In summary, all drugs developed and studied in clinical trials have efficiently reduced the risk of major adverse cardiovascular events (MACE), as well as benefits relative to risk reduction in heart-related events independently of the structural nature of the GLP-1R agonist [99]. On the other hand, studies in animal models have also provided important mechanisms behind the CVD prevention exerted by these class drugs [26]. One limitation of the GLP-1R analogues is the subcutaneous route of administration which has been avoided in semaglutide under oral prescription.

In recent years, other GLP-1R targeted drugs have been developed to overcome some limitations of the existing drugs. One of these is glutazumab (GMA105), a specific GLP-1/anti-GLP-1R antibody fusion protein consisting of the human GLP-1 derivative molecule linked to a humanized GLP-1R antibody via a peptide linker. This drug has been studied in preclinical phases [100] and has completed phase I clinical trials. Glatazumab is expected to overcome some limitations such as a better long-lasting effect and reduced toxicity. Danuglipron, also known as PF06882961, is another small oral molecule with GLP-1R agonist activity that has been proved in phase 1 clinical trials with T2DM subjects (NCT03538743) with successful results in relation to safety and tolerability and no clinically relevant electrocardiograms findings [101].

Unlike GLP-1R, GIPR agonism in monotherapy has not been so successful in the preclinical steps which halted the follow-up in clinical trials mostly due to the conflicting role of GIP in glycemic control [33]; therefore, no clinical trials with humans were successfully completed. However, as mentioned before, different studies have spotted GIPR agonism in combination with other GPCR ligands as a promising therapeutic strategy using unimolecular peptides with dual or triagonism. These strategies have reached clinical trial phases described below.

### 5.2. Studies in Humans with Dual Agonists 

In recent years, emergent therapies based on dual and triagonist activity targeted to the class B GPCR family members GLP-1R, GIPR, and GCGR have been developed for human clinical study [25]. Globally, these drugs display an enhanced effect in animal models when compared to those observed in animals treated with mono-agonists when studied in diabetic and obesity conditions. Table 1 summarizes the main clinical trials at advanced phases of these drugs, and, when available, the effects on CVD-related parameters are also shown.

#### 5.2.1. Clinical Trials Studying GLP-1R/GIPR Dual Agonists

Among all the GLP-1R/GIPR agonist candidates studied in pre-clinical stages, tirzepatide (LY3298176), is emerging as the lead from phase 1–2 clinical trials. SURPASS phase 2 clinical trials (1–6, COVT, J mono, J combo, and AP combo) in which tirzepatide is being used in different scenarios, were analyzed jointly regarding the cardiovascular event risk [102]. For all SURPASS trials, the primary endpoint was the mean change in HbA1c from baseline at 40–52 weeks, and the key secondary endpoint was the mean reduction in body weight from baseline at 40–52 weeks summarized in the COVT summit of 2021 [25]. In the meta-analysis performed by Sattar and colleagues [99,103], a comparison of the time of occurrence for MACE-4, which included four components within the trials, was made among treatments, with the confirmation of no increased cardiovascular risk. Notably, the SURPASS clinical trials, SURPASS-4 [104] and SURPASS-CVOT, whose estimated completion date is October 2024 [105], demonstrated the cardioprotective and safety effects of tirzepatide with primary endpoints for the safety of adjusted MACE events such as CV death, MI, and stroke, compared with insulin glargine in the SURPASS-4 and with dulaglutide in the SURPASS-CVOT trial.

Tirzepatide used once weekly subcutaneously is also being under deeper study in phase 3 SURMOUNT clinical trials (1–4, J, CN, OSA, Table 1) in people with diabetes and obesity. The main objective of this study is to gain more insight into the metabolic effects of the dual agonist and the long-lasting effect in the presence of other interventional lifestyle change approaches, such as a low-calorie diet or increased physical activity in people with obesity or overweight with or without T2DM and other related comorbidities. In these trials, only systolic and diastolic blood pressure are measured as secondary outcomes. Until now, only SURMOUNT-1, whose primary outcomes are body reduction from the baseline and the percentage of participants achieving ≥5% body weight reduction by week 72, reported results that indicate an improvement in all the pre-specified cardiometabolic parameters [106].

There are two more phase 3 clinical trials in which tirzepatide was also used. The SUMMIT trial was focused on studying the effect of tirzepatide in subjects with heart failure, preserved ejection fraction, and obesity (NCT04847557) without any results being posted yet (Table 1). The clinical trial NCT03861039 evaluated the long-term safety of the drug in combination with oral anti-hyperglycemic medications (sulfonylureas, biguanide, alpha-glucosidase inhibitor, thiazolidinedione, glinide, or sodium-glucose cotransporter type 2 inhibitor) in participants with T2DM. The study reported less than 1.5% of cardiac effects reported as Serious Adverse Events defined as death, hospitalization, or significant disability/incapacity among others, reaffirming the safety in terms of cardiac complications of the drug (https://clinicaltrials.gov/ct2/show/results/NCT03861039, accessed on 12 August 2022). The other clinical trial, NCT05260021, was focused on the paediatric population and it is estimated to end in December 2027.

Other GPL-1R/GIPR dual agonists have been developed [107]. Although some of them are on phase 1 or phase 2 clinical trials such as CT388, CT868, AMG133, or NN97097, none of them have posted or published any results (Table 1).

#### 5.2.2. Clinical Trials Evaluating GLP-1R/GCGR Dual Agonists

Regarding the GLP-1R/GCGR dual agonists, the most relevant GPL-1R/GCGR agonists developed in recent years are MK8521, SAR425899, and MEDI0382 or cotadutide. MK8521 had been studied in four different clinical trials including one focused on hypertension (NCT01446003), and although all of them have posted the outcomes on clinicaltrials.gov, there are no publications describing the results. SAR425899 was studied in six different clinical trials and, from phase 1 trials, it has been reported that it is safe and had enhanced the postprandial glycaemic control as liraglutide and improved the principal glycemic outcomes [108]. Finally, cotadutide has been studied in about 20 clinical trials, whose results have been reviewed in a recent meta-analysis that screened 663 relevant articles and included 8 investigations [109]. The global conclusion was a confirmation of the drug safety and effectiveness of the hypoglycemic drug at reducing body weight and HbA1c levels in patients with T2DM. Interestingly, cotadutide reduced hepatic steatosis, showing a benefit on hepatic inflammation and fibrosis markers yet surprisingly decreased hepatic glycogen. Cotadutide reduced blood pressure and augmented heart rates, which is attributed to the effect of the dual agonist in the heart and the vascular system, but these effects were similar to changes found for other GPL-1 analogues such as liraglutide.

There are other several ongoing phase 1 and phase 2 clinical trials [107,110] that have less information about efficacy. The NNC9204-1177 agonist, also called NN9277, was successful in reducing body weight in overweight and obese subjects versus placebo. However, NNC9204-1177 treatment was associated with safety issues including increased heart rate and liver damage markers and impaired glucose tolerance which prevents its further development for clinical use [111]. The same results were obtained with JNJ-6456511, also known as HM12525A, efinopegdutide, or MK6024-01. However, despite the significant reduction in the body weight of obese subjects without T2DM, the treatment provoked a greater incidence of adverse events [112]. Despite this, JNJ-6456511 is currently being evaluated in an ongoing phase 2 trial that ends on October 2022 in subjects with NASH (NCT04944992) to test the efficacy of reducing liver fat content compared with semaglutide. BI456906, a peptide that is being developed by Boehringer Ingelheim’s, was studied in a phase 1 clinical trial which confirmed its safety and tolerability, and now is undergoing phase 2 clinical trials in T2DM patients (NCT04153929) and overweight/obese patients (NCT04667377) to study the optimal dose and the efficiency of the drug but has no results posted. OPK88003, also called pegapamodutide, has posted results from its clinical trial (NCT03406377) but these have not yet been published. Regarding these later clinical trials, it seems that there is an increase in severe adverse effects when subjects are treated with these later dual agonists compared with the placebo group; however, there are no published results of finalized trials, and no information is available for their impact in coronary artery disease or myocardial infarction.

**Table 1:** Clinical trials of GPCR dual and triagonists.

Peptide-Derived Analogue	Clinical Trial Name and Registration Number	Phase	Subjects Studied and Treatments	CV Risk Reported Effects	References
GLP-1/GIP	SURPASS-1 (NCT03954834)	3	Drug-naïve people with T2DM/tirzepatide subcutaneous vs. placebo	No association with increased CV risk	[99,103,113]
	SURPASS-2 (NCT03987919)	3	People taking metformin/tirzepatide subcutaneous vs. semaglutide	No association with increased CV risk	[65,99,103]
	SURPASS-3 (NCT03882970)	3	People taking metformin with/without an SGLT2 inhibitor/tirzepatide subcutaneous vs. insulin degludec	No association with increased CV risk	[99,103,114]
	SURPASS-4 (NCT03730662)	3	T2DM patients with combinations of oral hypoglucemiants body-mass index >25 kg/m^2^ and CVD or CV risk Tirzepatide subcutaneous vs. insulin glargine	No association with increased CV risk over an extended follow-up of 104 weeks	[99,103,104]
	SURPASS-5 (NCT04039503)	3	Individuals taking insulin glargine with or without metformin. Tirzepatide subcutaneous vs. placebo	No association with increased CV risk	[99,103,115]
	SURPASS-6 (NCT04537923)	3	Insulin glargine treated subjects, with or without metformin. Tirzepatide vs. Insulin lispro	No association with increased CV risk	[99,103]
	SURPASS-CVOT (NCT04255433)		T2DM and atherosclerotic disease, and overweight. Tirzepatide subcutaneous vs. dulaglutide	No association with increased CV risk	[99,116]
	SURPASS J-mono (NCT03861052)		Japanese individuals who are drug-naïve or taking monotherapy. Tirzepatide vs. Dulaglutide		Not yet published
	SURPASS J-combo (NCT03861039)		Japanese people taking antidiabetes medications other than incretin-based classes. Tirzepatide vs. none	Less than 1% has SAEs related to cardiac disorders at doses above 10 mg and between 1.5–3.5% regarding AEs dose-dependent	Not yet published
	SURPASS AP-combo (NCT04093752)		Subjects from Australia, China, India, and the Republic of Korea taking metformin with/without a sulfonylurea Tirzepatide vs. Insulin glargine		Not yet published
	SURMONT-1 (NCT04184622)	3	Subjects with obesity or BMI 27 kg/m^2^ and related comorbidities. Tirzepatide vs. placebo.	Improvements in all prespecified cardiometabolic measures	[106]
	SURMONT-2 (NCT04657003)	3	Subjects with T2DM and body mass index of ≥27 kg/m^2^. Tirzepatide vs. placebo.		Not yet published
	SURMONT-3 (NCT04657016)	3	Subjects with obesity or body mass index of ≥27 kg/m^2^ and related comorbidities. Tirzepatide vs. placebo		Not yet published
	SURMONT-4 (NCT04660643)	3	People with obesity or body mass index of 27 kg/m^2^ and related comorbidities. Tirzepatide vs. placebo		Not yet published
	SURMOUNT J (NCT04844918)	3	Japanese people with a body mass index of ≥35 kg/m^2^ and at least one related comorbidity or of 27 –< 35 kg/m^2^ with two comorbidities. Tirzepatide subcutaneous vs. placebo		Not yet published
	SURMOUNT CN (NCT05024032)	3	Chinese subjects with a body mass index of 28 kg/m^2^ or 24 kg/m^2^ with related comorbidities. Tirzepatide subcutaneous vs. placebo		Not yet published
	SUMMIT (NCT04847557)	3	Obesity tirzepatide vs. placebo		Not yet published
	NCT03861039	3	T2DM patients Tirzepatide + antihyperglycemic medication	Less than 1.5% of SAEs,	Not yet published
GLP1/GCG	JNJ-64565111 (NCT03586830)	2	Individuals with T2DM and class II/III obesity JNJ-64565111 (different doses) vs. placebo	Not reported	[112]
	SAR425899 (NCT02973321)	2	Overweight to obese subjects with T2DM	Not reported	[108]
	Cotadutide (NCT04515849)	2	Participants who have chronic kidney disease with T2DM. Cotadutide (different doses) vs. placebo/semaglutide		Not yet published
	Cotadutide (NCT05364931) (PROXYMO-ADV)	2/3	Adult participants with non-cirrhotic and non-alcoholic steatohepatitis with fibrosis. Cotadutide vs. placebo		Not yet published
	Cotadutide (NCT03555994)	2	Overweight and obese subjects with T2DM Cotadutide vs. placebo/liraglutide		Not yet published
	Cotadutide (NCT03235050)	2	Overweight and obese subjects with T2D Cotadutide (different doses) vs. placebo/liraglutide	SAEs in all groups <1% and those related to CV seems to be slightly higher (but below 1%) in high-cotadutide dose groups	Not yet published
	Cotadutide (NCT04019561)	2	Obese subjects with NAFLD/NASH cotadutide low/high dose vs. placebo	CV-related SAEs around 4% in treatment group and 2–3x AEs in the same group compared to placebo but not CV-related	Not yet published
GPI/GLP1/GCG	SAR441255 (NCT04521738)		Lean-to-overweight healthy subjects SAR441255 vs. placebo	No significant changes in blood pressure or in the electrocardiogram parameters	[76]
	HM15211 (NCT03744182)	1	Obese subjects with NAFLD HM15211 vs. placebo	Not reported	[107]
	HM15211 (NCT04505436)	2	Subjects with biopsy confirmed NASH HM15211 vs. placebo	Not reported	[107]

### 5.3. Studies and Clinical Trials with GPL-1R/GIPR/GCGR Triagonists

Currently, only one molecule targeting the main preproglucagon-derived peptide family receptors, GLP-1R, GIPR, and GCGR is being further developed for clinical evaluation. Others such as NAR423 or NNC902-1706 have not moved forward in their development. There are some relevant data in the preclinical setting of SAR441255, which is being used in a clinical trial currently ongoing (NCT04521738), to test the safety of the drug [76]. It is expected that the cardiometabolic risk is not affected as it occurs in dual agonists; however, due to the wide targeting range of this compound, more focused trials in MACE-4 events should be performed in the future.

On the other hand, the novel long-acting HM15211 triagonist, which has been studied for safety and tolerability in obese patients, has also been considered for evaluation in two other clinical trials to study the effect on NASH (NCT03744182, NCT04505436), but no results have yet been posted or published [107].

## 6. Conclusions

Increasing evidence strongly indicates that T2DM and obese subjects develop hormone resistance and diminished GPCR-dependent signaling mediated by the three preproglucagon-derived hormones, glucagon, GLP-1, and GIP. On the other hand, one of the major mechanisms of the great success of bariatric metabolic surgery in T2DM and obese subjects is the restored metabolic control under the coordinated action of these hormones which spots the dual and triagonism of these pathways as logical therapeutic approaches. On top of this, there are two main reasons to pursue these strategies. The first reason is the success of the GLP-1 analogues and of the DPP4 inhibitors which not only ameliorate T2DM but also dramatically reduce macrovascular complications and CV events. The second reason is better knowledge of the coordinated action of the glucagon hormone and of the complexity of the GIP biology in islet cellular tone, which points to the need to restore their activity in order to achieve metabolic homeostasis. Therefore, the most recent drug developments are focused on the generation of unimolecular GPCR dual and triagonists and on the study of their effects in different preclinical and clinical investigations. Some of these dual and triagonists have been demonstrated to be safe and to display potential beneficial effects in the CV system. Hence, these therapeutic strategies hold great hope for the treatment of cardiometabolic diseases whose efficacy could be unprecedented. Further studies using preclinical models of CVD and the results of the ongoing clinical trials summarized here are needed in the following years to evaluate the real potential of these emergent therapies.

## Data Availability

All data are available from the corresponding author upon reasonable request.
